# Kidney Transplant Wait Times Under Waiting List Expansion Scenarios

**DOI:** 10.1001/jamanetworkopen.2025.1665

**Published:** 2025-03-24

**Authors:** Jillian S. Caldwell, Xingxing S. Cheng, Glenn M. Chertow, Jeremy D. Goldhaber-Fiebert

**Affiliations:** 1 School of Medicine, Division of Nephrology, Stanford University, Stanford, California; 2 Department of Health Policy, Stanford University, Stanford, California

## Abstract

**Importance:**

Kidney transplantation offers survival benefits and superior quality of life compared with maintenance dialysis for patients with end-stage kidney disease (ESKD), but it is limited to approximately 25 000 patients annually in the United States. Expanding access to transplant could be accomplished by allowing more patients to join the waiting list or by increasing organ supply.

**Objective:**

To estimate how these interventions could affect transplant wait times.

**Design, Setting, and Participants:**

This decision-analytic study used a Markov model with a simulated cohort of transplant-eligible US patients over 10 years (2022-2032).

**Exposures:**

Three expansion strategies: waiting list expansion alone, waiting list expansion with deceased-donor transplant (DDT) expansion, and waiting list expansion with living-donor transplant (LDT) expansion. Relative 10%, 25%, 50%, and 100% expansions of the current deceased-donor organ supply and 25%, 50%, 100%, and 200% expansions of current living donation rates were simulated, modeling 2 degrees of waiting list expansion (10% and 50%) for each strategy.

**Main Outcomes and Measures:**

Median wait time to kidney transplant using Kaplan-Meier survival analysis.

**Results:**

There were a total of 662 190 transplant-eligible patients in the simulated cohort, with a mean (SD) age of 58.7 (14.7) years; 327 126 (49%) female individuals; and 269 082 (41%) Black, 163 028 (25%) Hispanic, 233 739 (35%) non-Hispanic White, and 78 496 (12%) Asian individuals and individuals with another race or ethnicity. Under the baseline strategy, median (IQR) wait time was 32.8 (13.1-66.4) months and increased to 36.8 (14.7-74.7) months and 52.6 (21.0-107.9) months for 10% and 50% waiting list expansion alone. DDT and LDT expansion strategies added 1911 to 20 035 organs. For 10% waiting list expansion, median (IQR) wait times ranged from 23.7 (9.3-47.8) months to 34.5 (13.9-74.7) months. For 50% waiting list expansion, median (IQR) wait times ranged from 34.2 (13.6-69.4) months to 49.4 (19.7-101.0) months.

**Conclusions and Relevance:**

In this decision-analytic model, expansion strategies without additional organ supply increased the median time to kidney transplant by nearly 2 years; 10% waiting list expansion required at least 2850 additional organs to shorten wait times. However, 50% waiting list expansion required at least 11 000 additional organs to approximate current wait times. Reduction in the deceased-donor organ nonuse rate alone is unlikely to meet the shortfall. Systems-level efforts to significantly increase deceased and living donation should be prioritized alongside increased access to the transplant waiting list.

## Key Points

**Question:**

How might kidney transplant wait times change with a longer transplant waiting list and additional organ supply?

**Findings:**

Using a decision-analytic model with a simulated cohort of 662 190 transplant-eligible patients, adding 10% or 50% more patients to the waiting list was estimated to lengthen wait times by 4 to 20 months. Additional organs mitigated this increase: wait times could be maintained or shortened by adding 2800 kidneys (10% longer waiting list) or 11 000 kidneys (50% longer waiting list).

**Meaning:**

In this study, a longer waiting list prolonged wait times without additional organs; a substantial increase in all donation was required to maintain or shorten wait times.

## Teaser

This decision analytic model evaluates time to kidney transplant under different scenarios of expanding waiting lists, deceased-donor transplants, and living-donor transplants.

## Introduction

Kidney transplantation is considered the gold-standard treatment for kidney failure (end-stage kidney disease [ESKD]), offering survival benefits and a superior quality of life compared with dialysis for most patients.^1,2^ Despite these advantages, the availability of transplantation remains limited in the United States to approximately 25 000 kidney transplants annually.^3^ Although rates of deceased-donor transplantation (DDT) have risen in the past decade, from approximately 10 to 20 transplants per 100 patient-years, rates of living donor transplantation (LDT) remain stagnant.^3^ With limited organ supply, kidney transplant waiting lists have historically been restricted to healthier patients because the widespread availability of dialysis provides an alternative to transplantation, albeit an inferior one. Relatively few patients with ESKD are on the kidney transplant waiting list (12%-18% of the more than 500 000 patients receiving maintenance dialysis); yet, wait times are exceptionally long for those patients.^4^

Estimating current wait times is challenging due to the competing risks of waiting list removal (due to deteriorating health status for example) and death among transplant candidates. In fact, the median wait time in the presence of these competing risks has not been calculable for more than a decade because fewer than 50% of patients wait-listed 10 years ago have received a transplant.^5^ According to the US Renal Data System Annual Data Report (USRDS ADR), the estimated 2016 median wait time was 3.2 years counted from the time of listing to transplantation, censoring for death or loss to follow-up.^4^ Another approximation using a period-prevalent cohort of wait-listed patients from 2015 to 2018 and Kaplan-Meier survival analysis reported a median wait time of 3.8 years.^6^ The authors acknowledge that this method is limited by the assumption that all patients in the cohort will eventually receive a transplant.

Although overall organ availability falls far short of demand, a desire to extend the benefits of transplantation to additional patients has led to efforts to allow more patients to join the waiting list, alongside initiatives to increase organ supply. The effect of these interventions on transplant wait times has not been explored. Understanding how wait times might change under modifications to the current system is highly policy relevant. We developed a simulation model of patients with chronic kidney disease (CKD) and ESKD to estimate changes in wait times under various expansion scenarios involving waiting list additions with or without concomitant increases in DDT and LDT.

## Methods

### Ethics

This study was approved by the Stanford University institutional review board. Patient-level informed consent was not required, as data were deidentified national registry data. We adhered to the Consolidated Health Economic Evaluation Reporting Standards (CHEERS) reporting guidelines for health economics evaluations.

### Simulation Model

We constructed a decision-analytic Markov model to simulate a cohort of adult patients with CKD eligible for transplantation (estimated glomerular filtration rate [eGFR] ≤20mL/min/1.73 m^2^) and with ESKD receiving dialysis over a 10-year period (2022-2032). The cohort comprised 662 190 patients, our estimate of the number of adults living in the United States with non–dialysis dependent CKD and eGFR of 20mL/min/1.73 m^2^ or less and with ESKD receiving dialysis. The primary outcome of the model was median wait time. Patients younger than 18 years were excluded as they are prioritized differently in allocation schema, resulting in shorter wait times. Patients older than 80 years were excluded due to low rates of eligibility for transplantation. To reflect differences in mortality and transplantation rates, the cohort was subdivided into 3 age groups: 18 to 44 years, 45 to 64 years, and 65 to 79 years.

**Figure 1.**
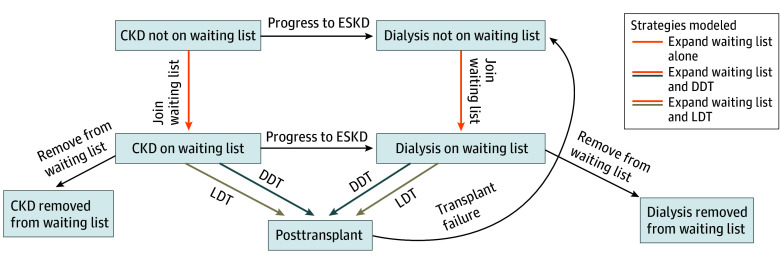
Markov Decision Model Depicting Waiting List and Organ Expansion Strategies Patients may begin the model with chronic kidney disease (CKD) or end-stage kidney disease (ESKD) and may start either on or off the waiting list. Patients with CKD may progress to ESKD. Patients on the waiting list may undergo deceased or living donor transplantation. Patients off the waiting list may also undergo living donor transplantation (arrow omitted for simplicity). Patients may be removed from the transplant waiting list and do not have a chance to return to the waiting list. After transplant, patients may experience transplant failure, requiring them to return to dialysis, where they have the chance to be relisted for transplant. Patients may either remain in the same state or die at each stage of the model (arrows omitted for simplicity). Expanding the waiting list (orange arrows) involves increasing the waiting list at model start by 10% or 50% and adding 10% or 50% more patients to the dialysis waiting list annually. Adding deceased-donor transplants (DDT; blue arrows) increases DDT by a relative 10%, 25%, 50%, or 100%. Adding living-donor transplants (LDT; tan arrows) increases LDT by a relative 25%, 50%, 100%, or 200%.

A simplified depiction of the model’s structure can be seen in Figure 1. Patients began on or off the waiting list, and each month, those not on the waiting list had a chance of being listed for DDT, undergoing LDT, remaining on dialysis, or dying. Patients with CKD had a chance of progressing to ESKD and beginning dialysis. Those on the waiting list had a chance of receiving a DDT, remaining wait-listed, being removed from the waiting list, or dying. Under living donor expansion scenarios, wait-listed patients also had a chance of undergoing LDT. After waiting list removal, patients experienced a monthly chance of dying; they could not be relisted for transplant. At transplant, patients could experience perioperative mortality or survive with graft function. Kidney transplant recipients could live with intact graft function, experience graft failure, or die. After graft failure, patients resumed dialysis and could be relisted for transplant. For simplicity, we assumed that patients could receive a DDT at any time on the waiting list and did not model active or inactive wait times. We excluded the possibilities of delayed graft function and primary nonfunction following kidney transplant.

### Waiting List Expansion Scenarios

We simulated the current transplant system (ie, status quo) and 3 expansion strategies: waiting list expansion alone, waiting list expansion with DDT expansion, and waiting list expansion with LDT expansion. We modeled 2 versions of each strategy: 10% and 50% waiting list expansion. Waiting list expansion involved starting 10% (or 50%) more patients on the waiting list at model start and adding 10% (or 50%) more patients to the waiting list per year. Expansion was applied to the CKD and ESKD waiting lists equally. Under expansion-alone strategies, the number of transplants occurring annually was unchanged from the status quo to reflect a constant rate of DDT and LDT.

To model DDT expansion, we increased their number by 10%, 25%, 50%, and 100% per year while keeping LDT constant. We modeled that 10% and 25% DDT expansion could be accomplished by reducing kidney discards (the 2022 proportion of unused kidneys was 26.7%)^7^; 50% and 100% DDT expansion would represent enhanced organ procurement up to a hypothetical upper limit of the potential deceased-donor organ pool (eTable 1 in Supplement 1). To model LDT expansion, we increased the number of annual transplants by 25%, 50%, 100%, or 200%, while keeping DDT constant. LDT expansion strategies incorporated a higher upper limit, as the maximum potential living donor pool is unknown.

In our model, patients receiving LDT under the status quo and DDT expansion strategies did not join the waiting list prior to transplantation because they typically wait less than 1 year for transplant.^8^ In contrast, the LDT expansion strategy aimed to simulate a hypothetical policy change newly incentivizing LDT, so under LDT expansion strategies, patients could undergo LDT while on the waiting list.

### Clinical Inputs

Details regarding clinical inputs and assessment of model face validity are in the eMethods in Supplement 1. Briefly, prevalence of transplant-eligible patients with CKD was derived from National Health and Nutrition Examination Survey (NHANES) 2017 to March 2020 data. Waiting list prevalence, probabilities of waiting list addition, removal, DDT, and LDT were derived from a cohort of individuals wait-listed for kidney-only transplant in 2022, using the Scientific Registry of Transplant Recipients (SRTR) database. Probabilities of progression to ESKD and posttransplant graft failure were derived from the 2021 USRDS ADR, while mortality rates were derived from the 2019 ADR to exclude mortality related to the COVID-19 pandemic. Parameter inputs are listed in eTable 2 in Supplement 1.

### Model Details

The Markov decision model used a monthly cycle length over a 10-year time horizon. We adopted a societal perspective and did not incorporate health utilities, costs, or discounting.

### Statistical Analysis

The primary outcome of the model was median wait time from listing to DDT or LDT. Wait time was derived using Kaplan-Meier product limit estimates to estimate total time on the waiting list.^6^ We did not perform statistical tests to compare wait times among strategies. Modeling and analysis were completed using TreeAge Pro version 2024 (TreeAge Software) and SAS version 9.4 (SAS Institute).

## Results

### Comparison With Published Data

The cohort consisted of 662 190 patients, with a mean (SD) age of 58.7 (14.7) years, and included 327 126 (49%) female individuals and 53 713 (8%) Asian, 269 082 (41%) Black, 163 028 (25%) Hispanic, and 233 739 (35%) non-Hispanic White individuals as well as 24 783 (4%) individuals with another race or ethnicity. Using inputs reflecting 2016 rates of transplantation as well as waiting list additions, removals, and deaths, median (IQR) wait time produced by our model was 48.6 (19.0-101.2) months compared with 48.2 (21.9-77.2) months in a cohort of patients wait-listed from 2016 to 2022 using SRTR data (eTable 3A in Supplement 1). This resembles other published estimates using period-prevalent cohorts (38.9 months^4^ and 45.2 months^6^). Survival probabilities can be seen in the eFigure in Supplement 1. Model outputs including counts of DDT, LDT, waiting list additions and removals, deaths occurring on the waiting list, and deaths while receiving dialysis were within 0.3% of outcomes published by the SRTR and USRDS (eTable 3B in Supplement 1).

### Status Quo

**Figure 2.**
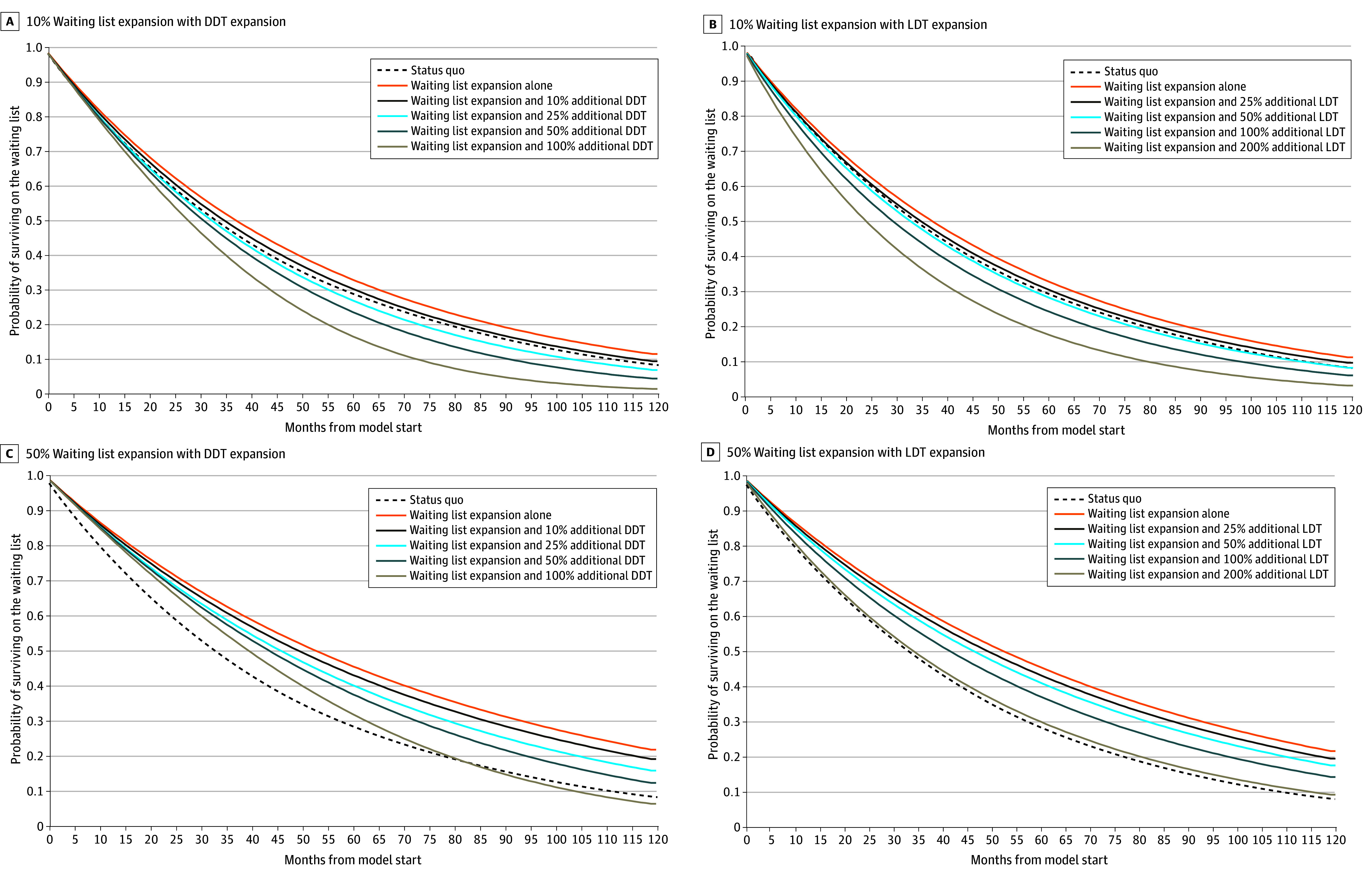
Kaplan-Meier Curves for the Probability of Surviving on the Waiting List for All Modeled Strategies Compared With the Status Quo A, Waiting list expansion of 10% with additional deceased-donor transplants (DDT). B, Waiting list expansion of 10% with additional living-donor transplants (LDT). C, Waiting list expansion of 50% with additional DDT. D, Waiting list expansion of 50% with additional LDT. Status quo (dashed line) shown in all panels for reference. Survival probabilities span a 10-year time horizon (2022-2032).

With inputs reflecting 2022 rates of transplantation, waiting list additions, removals, and deaths, model outputs after 1 year were within 0.5% of published outcomes (Table 1). Median (IQR) wait time produced by the status quo model was 32.8 (13.1-66.4) months (Figure 2).

**Table 1.** Status Quo and Expansion-Only Strategies^a^

Outcome	SRTR/USRDS outcomes (2022)	Status quo	Waiting list expansion	
			10%	50%
MWT (IQR), mo	NA	32.8	36.8 (14.7-74.7)	52.6 (21.0-107.9)
Change in MWT from status quo, mo	NA	NA	4.0	19.8
DDT, No.	19 944	19 938	19 947	19 946
LDT, No.	5660	5684	5684	5676
Waiting list additions, No.	33 215	33 200	36 746	49 653
Waiting list removals, No.	6040	6031	6709	9330
Deaths occurring on the waiting list	4454	4478	4634	6892

Abbreviations: DDT, deceased donor transplant; LDT, living donor transplant; MWT, median wait time; NA, not applicable; SRTS, Scientific Registry of Transplant Recipients; USRDS, US Renal Data System.

^a^ Model outputs after 1 year for the status quo and waiting list expansion-only strategies.

### Waiting List Expansion Alone

Under expansion-alone strategies, median (IQR) wait times increased to 36.8 (14.7-74.4) months and 52.6 (21.0-107.9) months for 10% and 50% waiting list expansion, respectively (Table 1 and Figure 2). Numbers of DDT and LDT were unchanged, by design. Waiting list additions and removals as well as deaths occurring on the waiting list increased relative to the status quo.

### Waiting List Expansion and DDT Expansion

A 10% to 100% relative DDT expansion resulted in between 1911 and 20 035 additional transplants (Table 2). With 10% waiting list expansion and 10% DDT expansion, median (IQR) wait time was 34.5 (13.9-69.1) months, 2 months longer than the status quo. Expanding DDT by 25% to 100% shortened wait times relative to the status quo by 0.8 to 8.4 months (Figure 2A).

**Table 2.** Waiting List Expansion With DDT Expansion^a^

Outcome	DDT expansion rate							
	10% Waiting list expansion				50% Waiting list expansion			
	10%	25%	50%	100%	10%	25%	50%	100%
MWT (IQR), mo	34.5 (21.1-107.9)	32.0 (13.1-62.8)	30.4 (12.8-57.5)	24.4 (12.1-48.7)	49.2 (19.9-99.5)	46.7 (18.7-90.4)	43.4 (18.3-82.4)	39.3 (19.4-70.0)
Change in MWT from status quo, mo	1.7	−0.8	−2.4	−8.4	16.7	13.9	10.6	6.5
DDT, No.	21 858	24 955	29 691	39 928	21 913	24 948	29 794	39 982
LDT, No.	5685	5685	5685	5686	5676	5676	5677	5677
Waiting list additions, No.	36 745	36 746	36 747	36 750	49 654	49 655	49 657	49 662
Waiting list removals, No.	6656	6569	6434	6131	9277	9193	9058	8769
Deaths occurring on the waiting list, No.	4593	4527	4424	4194	6848	6779	6668	6429

Abbreviations: MWT, median wait time; DDT, deceased-donor transplant; LDT, living-donor transplant.

^a^ Model outputs after 1 year for strategies simulating waiting list expansion with DDT expansion.

With 50% waiting list expansion, all scenarios resulted in a longer wait time than the status quo. Wait times ranged from a median (IQR) of 49.2 (19.9-99.5) months to 39.3 (19.4-70.0) months (Table 2 and Figure 2C). In all scenarios, waiting list removals were greater than the status quo. For most scenarios, deaths occurring on the waiting list displayed a similar pattern to waiting list removals, but under the scenario of 10% waiting list expansion and 50% to 100% DDT expansion, fewer waiting list deaths occurred compared with the status quo.

### Waiting List Expansion and LDT Expansion

A 25% to 200% relative LDT expansion resulted in between 1414 and 11 365 additional transplants (Table 3). With 10% waiting list expansion and 25% LDT expansion, median (IQR) wait time was 34.5 months (13.8-70.0), 2 months longer than the status quo. Expanding LDT by 50% to 200% shortened wait times relative to status quo by 0.3 to 9.0 months (Figure 2B).

**Table 3.** Waiting List Expansion With LDT Expansion^a^

Outcome	LDT expansion rate							
	10% Waiting list expansion				50% Waiting list expansion			
	25%	50%	100%	200%	25%	50%	100%	200%
MWT (IQR), mo	34.5 (13.8-70.0)	32.5 (13.0-65.7)	29.0 (11.5-58.6)	23.7 (9.3-47.8)	49.4 (19.7-101.0)	46.5 (18.6-94.9)	41.6 (16.7-84.8)	34.2 (13.6-69.4)
Change in MWT from status quo, mo	1.7	−0.3	−3.8	−9.0	16.6	13.7	8.8	1.4
DDT, No.	19 943	19 947	19 959	19 956	19 940	19 947	19 950	19 956
LDT, No.	7098	8535	11 353	17 042	7118	8539	11 349	17 050
Waiting list additions, No.	36 743	36 743	36 744	36 746	49 707	49 708	49 709	49 707
Waiting list removals, No.	6657	6617	6538	6377	9276	9237	9160	9002
Deaths occurring on the waiting list, No.	4619	4591	4535	4422	6879	6850	6793	6676

Abbreviations: MWT, median wait time; DDT, deceased-donor transplant; LDT, living-donor transplant.

^a^ Model outputs after 1 year for strategies simulating waiting list expansion with LDT expansion.

With 50% waiting list expansion, median wait time was longer compared with the status quo in all hypothetical scenarios by 1.4 to 16.6 months (Table 3 and Figure 2D). Waiting list removals and deaths occurring on the waiting list were increased relative to the status quo, except under the scenario of 10% waiting list expansion and 200% LDT expansion, in which fewer deaths occurred compared with the status quo.

## Discussion

Our analysis, using a simulation model that can reproduce wait times from various analyses of US transplant data, broadly explores how wait times change with additions to the transplant waiting list and changes to the deceased and living donor organ supplies. We found that with waiting list expansions, median wait times would increase. We also found that substantial increases to either deceased or living transplantation, reflecting fundamental changes in the current models of organ procurement, would be required to maintain or reduce wait times.

Transplant wait times are important to patients with ESKD. Discrete choice experiments show that most patients prefer to accept lower-quality kidneys to reduce their wait times, rather than waiting for a higher-quality kidney.^9,10,11^ Furthermore, longer wait times and extended time receiving dialysis are linked to poorer posttransplant outcomes, underscoring the urgency of timely transplantation.^12,13^ We chose to approximate median wait time using Kaplan-Meier survival analysis, a method with limitations, but that allows us to explore how wait times could change under various expansion scenarios.

We found that waiting list expansion alone would prolong wait times by 4 months for a 10% expansion to nearly 20 months for a 50% expansion. The upper limit of patients who might benefit from transplantation is unknown, but estimates from other countries suggest that at least 50% of patients with ESKD may be eligible for transplantation.^14,15^ Our expansion strategies are relative to low rates of wait-listing overall: only 12% to 18% of patients receiving dialysis are currently listed for transplantation.^4^ With a relative expansion of the waiting list by 10% or 50%, the absolute percentage of patients receiving dialysis listed for transplant would increase from 12.4% to 13.9% or 19.6%, respectively. A recent study from dialysis units in the Southeast United States estimated that 21% to 25% of patients not referred for transplant may have been potentially good transplant candidates, recommending a 14% increase in transplant referrals from dialysis centers as a reasonable and achievable goal.^16^ Not all patients referred for transplant are wait-listed, but based on our findings, an absolute increase of merely 7% in wait-listed candidates could prolong wait times by nearly 2 years without concomitant increases in organ supply.

Our 10% waiting list expansion scenarios required more than 2800 additional organs to maintain or shorten current wait times, but 50% waiting list expansion resulted in longer wait times despite the addition of more than 11 000 additional organs. This highlights the degree of organ supply expansion required to keep pace with waiting list expansion. We estimated the upper limit of the potential deceased donor organ pool of approximately 40 000 kidneys per year—much higher than what could be achieved by merely eliminating nonuse of currently procured kidneys. In 2022, 7392 recovered kidneys (26.7%) were not transplanted, more commonly among older, more ill donors, for whom graft longevity may be limited compared with kidneys from younger donors.^3,7^ Comparisons between the United States and France reveal more aggressive offer acceptance in France, with one publication suggesting that up to 60% of discarded kidneys in the United States would have been transplanted under the French system.^17^ In contrast, a review by transplant physicians of organ offers from a single organ procurement organization (OPO) in California found that only 12% of nonutilized kidneys were potentially transplantable.^18^ Scrutiny of transplant center outcomes, such as 1-year graft survival, may disincentivize centers against more aggressive offer acceptance practices. The Organ Procurement and Transplantation Network (OPTN) has implemented a pretransplant offer acceptance rate ratio metric to encourage centers to accept more organ offers, but its effect remains to be seen.^19^

In our models, 10% and 25% relative increase in deceased donor organs corresponded to a reduction or near-elimination of kidney nonuse. These increases could maintain wait times with a 10% longer waiting list, but not with a 50% longer waiting list. Thus, reducing the discard rate alone is unlikely to meet the demands of waiting list expansion, and strategies must focus on increasing deceased donor organ procurement. One potential area for improvement is donation after cardiac death (DCD), which varies substantially among OPOs; percentages of DCD per OPO range from 30% to 53%. It is estimated that if all OPOs procured approximately 50% of donor organs from DCD, the number of DCD donors overall could be doubled (quadrupling the number of potential kidneys), bringing the total organ supply in line with our 25% to 50% DDT expansion strategies.^20^

In contrast, the upper limit of potential living donors is unknown. We modeled a wide range of increases in LDT to capture uncertainty regarding the feasibility of LDT expansion. Unfortunately, LDT rates have been stagnant over the past decade.^3^ Although many living donors may be willing, comorbidities such as diabetes and obesity, immunologic compatibility with recipients, or financial limitations for donors often preclude donation. The End Kidney Deaths Act, introduced in the House of Representatives, proposes refundable tax credits of $10 000 per year for 5 years to living kidney donors to diminish the financial burden of donation.^21,22^

Faced with longer wait times, patients and families might become more motivated to pursue living donor transplantation. Historically, recipients of LDT are more likely to be White, highly educated, and to have private insurance—characteristics linked with higher rates of wait-listing and transplantation.^23^ Waiting list expansion is often proposed as a method to alleviate racial, ethnic, and socioeconomic disparities in access to transplantation.^24^ Efforts to increase rates of LDT are under way in racial and ethnic minority groups, but an analysis of Black, Hispanic, and Asian patients between 1999 and 2014 revealed that disparities in LDT rates have worsened over time.^25,26^ Given these findings, there is a critical need to continue research and policy initiatives that promote living donation, particularly for patients of designated racial and ethnic minority groups and lower socioeconomic status.

We should acknowledge that waiting list additions and removals require a significant and costly effort on the part of transplant programs to coordinate. Some advocate for a change from the current opt-in waiting list system to an opt-out system in which patients are automatically referred for transplantation, arguing that this would streamline the referral process.^24^ However, triaging referrals, organizing transplant evaluations, and scheduling committee reviews requires adequate staffing and compensation, with costs predominantly shouldered by Medicare.^27^ Our model does not quantify systems-level increases in staffing and management required to enact waiting list expansion strategies.

We estimated increases in waiting list deaths concordant with waiting list additions. The OPTN’s new pretransplant mortality rate ratio^19^ is aimed to reduce waiting list mortality by incentivizing transplantation. We observed that even with increases in organ supply, adding more patients to the waiting list would likely result in more patients dying on the waiting list. Although the pretransplant mortality rate ratio metric incorporates risk adjustment and is normalized to national expectations, it may have the unintended consequence of penalizing transplant programs with high rates of waiting list additions.

### Limitations

Our approach has several limitations. Our model counted wait time from model start and excludes wait time accumulated by those who begin on the waiting list. We also did not distinguish active from inactive wait time (ie, time accrued without the potential to receive kidney offers).^28,29^ Additionally, because wait time is accrued from the first date a patient receives dialysis (not the date of first listing), some centers may list patients later in the course of disease. Our model counts wait time from date of listing, not date of first dialysis. These omissions likely bias our wait time estimates to be shorter than those experienced by patients. We did not account for differences in wait times among patients according to blood type and levels of preformed antibodies, which affect the number of potential matches between a patient and donor, or geographic location, due to differences in regional OPOs.^30,31^ Our model also did not incorporate specific allocation policies. Additionally, our model is limited by precision in parameter inputs. Although inputs were derived from rigorously collected data published by the USRDS and SRTR, fidelity may be lost under a simulation model. However, the wait time estimates derived from our model are not meant to apply to individual patients but rather to be interpreted relative to one another to convey the implications of various strategies for waiting list expansion. It is possible, especially for questions concerning the distributional equity effects of waiting list expansion and for resource planning in specific transplant settings, that a decision-analytic Markov modeling approach like the one we used to answer our specific research question may not be sufficient. Other modeling methods, including individual-level models like discrete event simulation and agent-based microsimulations or system dynamic models, may be preferrable to answer these questions.

## Conclusions

In this decision analytic model of transplant-eligible patients with kidney disease, we found that expanding the transplant waiting list would result in longer wait times that can only be alleviated by drastically increasing organ supply. Reducing the deceased donor nonuse rate is unlikely to meet the increasing demand; large increases in deceased donor procurement and/or living donation rates would be needed. Efforts to expand access to transplantation must consider the need for growth at all stages of the transplant process and should incorporate estimates of changes in median wait times to inform all affected stakeholders.

## Notes

Supplement 1.
eMethods.
**eTable 1. **Estimation of Upper Limit of the Deceased Donor Organ Pool **eTable 2. **Parameter Inputs **eTable 3. **Model Face Validity **eFigure. **Model Face Validity—Survival Probabilities
eReferences.

Supplement 2.
Data Sharing Statement
