# Supplementary material for: Kidney Transplant Wait Times Under Waiting List Expansion Scenarios
Source: JAMA Netw Open. 2025 Mar 24;8(3):e251665. doi: 10.1001/jamanetworkopen.2025.1665 (PMC11933994; doi:10.1001/jamanetworkopen.2025.1665)
Supplement: Supplement 1. — eMethods. eTable 1. Estimation of Upper Limit of the Deceased Donor Organ Pool eTable 2. Parameter Inputs eTable 3. Model Face Validity eFigure. Model Face Validity—Survival Probabilities eReferences. [file jamanetwopen-e251665-s001.pdf]

Supplemental Online Content

Caldwell JS, Cheng XS, Chertow GM, Goldhaber-Fiebert JD. Kidney transplant wait times under waiting list expansion scenarios. *JAMA Netw Open.* 2025;8(3):e251665.
doi:10.1001/jamanetworkopen.2025.1665

eMethods.

eTable 1. Estimation of Upper Limit of the Deceased Donor Organ Pool

eTable 2. Parameter Inputs

eTable 3. Model Face Validity

eFigure. Model Face Validity—Survival Probabilities

eReferences.

This supplemental material has been provided by the authors to give readers additional information about their work.

© 2025 Caldwell JS et al. *JAMA Network Open*.

eMethods.

Description of Data Sources

National Health and Nutrition Examination Survey (NHANES)

The NHANES is a program of studies designed to assess the health and nutritional status of adults and children in the United States. Survey findings are also the basis for national standards for such measurements as height, weight, and blood pressure[1]. Laboratory data including serum creatinine were used to estimate the number of patients with transplant-eligible CKD in the U.S. population.

Scientific Registry of Transplant Recipients (SRTR)

This study used data from the Scientific Registry of Transplant Recipients (SRTR). The SRTR data system includes data on all donor, wait-listed candidates, and transplant recipients in the U.S., submitted by the members of the Organ Procurement and Transplantation Network (OPTN). The Health Resources and Services Administration (HRSA), U.S. Department of Health and Human Services provides oversight to the activities of the OPTN and SRTR contractors. This dataset enabled us to derive parameter inputs for waitlist prevalence, waitlist additions, waitlist removals, and deceased and living-donor transplantation rates.[2]

United States Renal Data System (USRDS)

The USRDS is a national data system that collects, analyzes, and distributes information about end-stage renal disease (ESKD) in the United States. USRDS staff collaborate with staff from the Centers for Medicare & Medicaid Services (CMS), the United Network for Organ Sharing, and the ESKD networks, sharing data sets and actively working to improve the accuracy of ESKD patient information. USRDS maintains a stand-alone database on the diagnoses and demographic characteristics of ESKD patients, along with biochemical data, dialysis claims, and information on treatment and payer histories, hospitalization events, deaths, physician and supplier services, and providers.[3] USRDS data were used to derive parameter inputs for ESKD incidence, prevalence, ESKD and CKD mortality, progression to ESKD, post-transplant graft failure and post-transplant mortality.

© 2025 Caldwell JS et al. *JAMA Network Open*.

Derivation of Clinical Inputs

Prevalence of transplant-eligible patients with CKD was derived from National Health and Nutrition Examination Survey (NHANES) 2017-March 2020 data.[1] Serum creatinine concentrations were used to estimate glomerular filtration rates using the CKD-EPI 2021 equation.[4] Individuals with eGFR $\leq$ 20ml/min/1.73m^2 based on serum creatinine concentrations and who responded "No" or with a missing response to the Kidney Conditions survey question "In the past 12 months, have you received dialysis?" were counted as having non-dialysis dependent, transplant-eligible CKD. Prevalence of dialysis-dependent ESKD was derived from the USRDS ADR, which publishes data for all patients with ESKD.

Waitlist prevalence, probabilities of waitlist addition, removal, DDTx, and LDTx were derived from a cohort of individuals waitlisted for kidney-only transplant in 2022, using the Scientific Registry of Transplant Recipients (SRTR) database, which tracks all solid organ transplants in the U.S. Although patients can be listed for transplant at multiple centers, we counted waitlist additions and removals at the candidate level. Additions were only counted if the patient was not already listed at other centers. Removals were only counted if all listings for a particular candidate were removed. Removals were recorded if listed under the categories 'medically unsuitable', 'condition deteriorated' or 'other' (which may include removals for financial reasons, insurance complications, non-adherence, or psychosocial barriers). Data were missing in <1% of the SRTR cohort used to derive input parameters.

Probabilities of progression to ESKD and post-transplant graft failure were based on the 2021 USRDS ADR, while mortality rates were derived from 2019 USRDS ADR due to increased mortality during 2020 and 2021 because of the COVID-19 pandemic. Perioperative mortality rates were estimated from Ying et al.[5]

Probabilities of progression to ESKD, waitlist removal, graft failure, and peri-operative mortality were unchanged under expansion strategies. For patients with CKD, waitlisted and non-waitlisted patients had the same risk of death. For patients receiving dialysis, waitlisted patients had lower mortality than non-waitlisted patients, because the transplant wait list generally contains healthier patients. Based on previous research suggesting that up to 50% of non-waitlisted dialysis patients may have similar mortality rates to those currently waitlisted,[6,7] we did not change waitlist mortality for patients joining the waitlist in the 10% expansion strategy and patients age 18-44 in the 50% expansion strategy. For the 50% expansion strategy, we increased the waitlist mortality rate by 10% for the remaining age groups to reflect the inclusion of

© 2025 Caldwell JS et al. *JAMA Network Open*.

sicker patients on the waitlist. To account for a potentially sicker pool of non-waitlisted patients after moving these patients onto the waitlist, we adjusted the non-waitlist dialysis mortality rate upward to match total annual dialysis deaths.

Model Face Validity

To assess whether our model reasonably reflected survival probabilities and wait times experienced by candidates, we assembled a cohort listed for kidney-only transplant at any time between January 1, 2016, and August 31, 2022 using SRTR data. Because our model cannot account for the time spent waiting on the list prior to model start, we counted wait time beginning on the later of two dates: January 1, 2016 or the date of waitlisting. Candidates were censored at waitlist removal, death, or end of follow-up (August 31, 2022). 2.26% of the cohort was lost to follow-up. Kaplan-Meier survival analysis was used to compute median wait time. To compare the median wait time generated by this 2016 cohort to our model, we modified probabilities of receiving DDTx and LDTx, waitlist addition, waitlist removal and death to reflect 2016 rates from SRTR or USRDS as described above.

© 2025 Caldwell JS et al. *JAMA Network Open*.

eTable 1. Estimation of Upper Limit of the Deceased Donor Organ Pool

Variable	Value	Source	Notes
Total number of deaths occurring in 2019	2,854,838	CDC/National Center for Health Statistics 2020[8]	2019 deaths chosen to exclude deaths related to COVID-19
Potential organ donors	42,823	Klassen 2016[9]	Estimated 1.5% of all deaths. “Potential organ donor” defined as patient under 75 years, death occurring in-hospital, excluding malignancy, sepsis diagnoses codes, and limited to patients who are ventilated
Number of potential organ donors via DCD pathway	12,847	SRTR Annual Data Report 2022[10]	Estimated 30% of potential organ donors are DCD
Medically suitable DCD organ donors	3,854	Mone 2024[11]	Estimated 70% of potential DCD donors excluded due to medical reasons (e.g., age, organ dysfunction, suspected infection, hemodynamic instability)
Number of potential organ donors via DBD pathway	29,976	SRTR Annual Data Report 2022[10]	Estimated 70% of potential organ donors are DBD
Medically suitable DBD donors	26,978	Marsolais 2022[12]	Estimated 90% of potential DBD donors excluded due to medical reasons (e.g., age, organ dysfunction, infection, coagulopathy)
Total medically suitable DCD + DBD donors	30,832		
Number of potential donors consenting to organ donation	21,583	Goldberg 2013[13]	Estimate 70% of families approached for organ donation consent
Potential DDTx recovered	41,007	SRTR Annual Data Report 2022[10]	Estimate 1.9 kidneys recovered per donor

CDC, Centers for Disease Control; DBD, deceased after brain death; DCD, deceased after cardiac death; DDTx, deceased donor kidney transplant; SRTR, Scientific Registry of Transplant Recipients.

© 2025 Caldwell JS et al. *JAMA Network Open*.

eTable 2. Parameter Inputs

	Status Quo			10% Expansion			10% Expansion + 10% DDTx			10% Expansion + 25% DDTx			10% Expansion + 50% DDTx			10% Expansion + 100% DDTx			10% Expansion + 25% LDTx		
	Age 18-44	Age 45-64	Age 65-79	Age 18-44	Age 45-64	Age 65-79	Age 18-44	Age 45-64	Age 65-79	Age 18-44	Age 45-64	Age 65-79	Age 18-44	Age 45-64	Age 65-79	Age 18-44	Age 45-64	Age 65-79	Age 18-44	Age 45-64	Age 65-79
CKD, not on waitlist[a]	30.9%	22.5%	28.1%	30.9%	22.5%	28.1%	30.9%	22.5%	28.1%	30.9%	22.5%	28.1%	30.9%	22.5%	28.1%	30.9%	22.5%	28.1%	30.9%	22.5%	28.1%
ESKD, not on waitlist[b]	43.8%	59.2%	65.1%	41.3%	57.3%	64.4%	41.3%	57.3%	64.4%	41.3%	57.3%	64.4%	41.3%	57.3%	64.4%	41.3%	57.3%	64.4%	41.3%	57.3%	64.4%
CKD, on waitlist[c]	6.0%	4.9%	2.0%	6.6%	5.4%	2.2%	6.6%	5.4%	2.2%	6.6%	5.4%	2.2%	6.6%	5.4%	2.2%	6.6%	5.4%	2.2%	6.6%	5.4%	2.2%
ESKD, on waitlist[c]	19.3%	13.5%	4.8%	21.2%	14.8%	5.3%	21.2%	14.8%	5.3%	21.2%	14.8%	5.3%	21.2%	14.8%	5.3%	21.2%	14.8%	5.3%	21.2%	14.8%	5.3%
Progress to ESKD[b]	0.048	0.081	0.076	0.047	0.077	0.072	0.047	0.077	0.072	0.047	0.077	0.072	0.047	0.077	0.072	0.047	0.077	0.072	0.047	0.077	0.072
Add to WL, CKD[c]	0.011	0.015	0.006	0.012	0.016	0.006	0.012	0.016	0.006	0.012	0.016	0.006	0.012	0.016	0.006	0.012	0.016	0.006	0.012	0.016	0.006
Add to WL, ESKD[c]	0.013	0.006	0.002	0.015	0.006	0.002	0.015	0.006	0.002	0.015	0.006	0.002	0.015	0.006	0.002	0.015	0.006	0.002	0.015	0.006	0.002
Remove from WL, CKD[c]	0.003	0.005	0.007	-	-	-	-	-	-	-	-	-	-	-	-	-	-	-	-	-	-
Remove from WL, ESKD[c]	0.003	0.005	0.007	-	-	-	-	-	-	-	-	-	-	-	-	-	-	-	-	-	-
DDTx, CKD[c]	0.007	0.008	0.013	0.006	0.007	0.011	0.006	0.007	0.011	0.006	0.007	0.011	0.006	0.007	0.011	0.006	0.007	0.011	0.006	0.007	0.011
DDTx, ESKD[c]	0.018	0.016	0.021	0.016	0.015	0.019	0.018	0.016	0.021	0.022	0.019	0.025	0.027	0.024	0.031	0.039	0.035	0.045	0.016	0.015	0.019
LDTx, CKD[c]	0.082	0.059	0.059	0.082	0.059	0.059	0.082	0.059	0.059	0.082	0.059	0.059	0.082	0.059	0.059	0.082	0.059	0.059	0.082	0.059	0.059
LDTx, ESKD[c]	0.216	0.176	0.196	0.197	0.159	0.177	0.197	0.159	0.177	0.197	0.159	0.177	0.197	0.159	0.177	0.197	0.159	0.177	0.197	0.159	0.177
New LDTx, CKD	n/a	n/a	n/a	n/a	n/a	n/a	n/a	n/a	n/a	n/a	n/a	n/a	n/a	n/a	n/a	n/a	n/a	n/a	0.001	0.0009	0.001
New LDTx, ESKD	n/a	n/a	n/a	n/a	n/a	n/a	n/a	n/a	n/a	n/a	n/a	n/a	n/a	n/a	n/a	n/a	n/a	n/a	0.001	0.0009	0.001
Periop. death[d]	0.002	0.002	0.002	-	-	-	-	-	-	-	-	-	-	-	-	-	-	-	-	-	-
Death, CKD[b]	0.001	0.003	0.006	-	-	-	-	-	-	-	-	-	-	-	-	-	-	-	-	-	-
Waitlist death, ESKD[c]	0.003	0.004	0.004	0.003	0.004	0.004	0.003	0.004	0.004	0.003	0.004	0.004	0.003	0.004	0.004	0.003	0.004	0.004	0.003	0.004	0.004
Death, ESKD, not on waitlist[b]	0.006	0.011	0.017	0.006	0.011	0.017	0.006	0.011	0.017	0.006	0.011	0.017	0.006	0.011	0.017	0.006	0.011	0.017	0.006	0.011	0.017
Death, post-transplant[b]	0.001	0.002	0.003	-	-	-	-	-	-	-	-	-	-	-	-	-	-	-	-	-	-

© 2025 Caldwell JS et al. *JAMA Network Open*.

eTable 2. Parameter Inputs (continued)

	10% Expansion + 50% LDTx			10% Expansion + 100% LDTx			10% Expansion + 200% LDTx			50% Expansion			50% Expansion + 10% DDTx			50% Expansion + 25% DDTx			50% Expansion + 50% DDTx		
	**Age 18-44**	**Age 45-64**	**Age 65-79**	**Age 18-44**	**Age 45-64**	**Age 65-79**	**Age 18-44**	**Age 45-64**	**Age 65-79**	**Age 18-44**	**Age 45-64**	**Age 65-79**	**Age 18-44**	**Age 45-64**	**Age 65-79**	**Age 18-44**	**Age 45-64**	**Age 65-79**	**Age 18-44**	**Age 45-64**	**Age 65-79**
CKD, not on waitlist[a]	30.9%	22.5%	28.1%	30.9%	22.5%	28.1%	30.9%	22.5%	28.1%	30.9%	22.5%	28.1%	30.9%	22.5%	28.1%	30.9%	22.5%	28.1%	30.9%	22.5%	28.1%
ESKD, not on waitlist[b]	41.3%	57.3%	64.4%	41.3%	57.3%	64.4%	41.3%	57.3%	64.4%	31.2%	50.0%	61.7%	31.2%	50.0%	61.7%	31.2%	50.0%	61.7%	31.2%	50.0%	61.7%
CKD, on waitlist[c]	6.6%	5.4%	2.2%	6.6%	5.4%	2.2%	6.6%	5.4%	2.2%	8.9%	7.3%	3.1%	8.9%	7.3%	3.1%	8.9%	7.3%	3.1%	8.9%	7.3%	3.1%
ESKD, on waitlist[c]	21.2%	14.8%	5.3%	21.2%	14.8%	5.3%	21.2%	14.8%	5.3%	28.9%	20.2%	7.2%	28.9%	20.2%	7.2%	28.9%	20.2%	7.2%	28.9%	20.2%	7.2%
Progress to ESKD[b]	0.047	0.077	0.072	0.047	0.077	0.072	0.047	0.077	0.072	0.047	0.069	0.072	0.047	0.069	0.072	0.047	0.069	0.072	0.047	0.069	0.072
Add to WL, CKD[c]	0.012	0.016	0.006	0.012	0.016	0.006	0.012	0.016	0.006	0.017	0.021	0.008	0.017	0.021	0.008	0.017	0.021	0.008	0.017	0.021	0.008
Add to WL, ESKD[c]	0.015	0.006	0.002	0.015	0.006	0.002	0.015	0.006	0.002	0.025	0.010	0.003	0.025	0.010	0.003	0.025	0.010	0.003	0.025	0.010	0.003
Remove from WL, CKD[c]	-	-	-	-	-	-	-	-	-	-	-	-	-	-	-	-	-	-	-	-	-
Remove from WL, ESKD[c]	-	-	-	-	-	-	-	-	-	-	-	-	-	-	-	-	-	-	-	-	-
DDTx, CKD[c]	0.006	0.007	0.011	0.006	0.007	0.011	0.006	0.007	0.011	0.004	0.005	0.008	0.004	0.005	0.008	0.004	0.005	0.008	0.004	0.005	0.008
DDTx, ESKD[c]	0.017	0.015	0.019	0.017	0.015	0.019	0.018	0.015	0.020	0.012	0.011	0.014	0.013	0.012	0.015	0.015	0.014	0.018	0.019	0.017	0.022
LDTx, CKD[c]	0.082	0.059	0.059	0.082	0.059	0.059	0.082	0.059	0.059	0.063	0.044	0.045	0.063	0.044	0.045	0.063	0.044	0.045	0.063	0.044	0.045
LDTx, ESKD[c]	0.197	0.159	0.177	0.197	0.159	0.177	0.197	0.159	0.177	0.155	0.122	0.136	0.155	0.122	0.136	0.155	0.122	0.136	0.155	0.122	0.136
New LDTx, CKD	0.003	0.002	0.002	0.005	0.004	0.004	0.011	0.007	0.009	n/a	n/a	n/a	n/a	n/a	n/a	n/a	n/a	n/a	n/a	n/a	n/a
New LDTx, ESKD	0.003	0.002	0.002	0.005	0.004	0.004	0.011	0.007	0.009	n/a	n/a	n/a	n/a	n/a	n/a	n/a	n/a	n/a	n/a	n/a	n/a
Periop. death[d]	-	-	-	-	-	-	-	-	-	-	-	-	-	-	-	-	-	-	-	-	-
Death, CKD[b]	-	-	-	-	-	-	-	-	-	-	-	-	-	-	-	-	-	-	-	-	-
Waitlist death, ESKD[c]	0.003	0.004	0.004	0.003	0.004	0.004	0.003	0.004	0.004	0.003	0.005	0.004	0.003	0.005	0.004	0.003	0.005	0.004	0.003	0.005	0.004
Death, ESKD, not on waitlist[b]	0.006	0.011	0.017	0.006	0.011	0.017	0.006	0.011	0.017	0.008	0.012	0.018	0.008	0.012	0.018	0.008	0.012	0.018	0.008	0.012	0.018
Death, post-transplant[b]	-	-	-	-	-	-	-	-	-	-	-	-	-	-	-	-	-	-	-	-	-

© 2025 Caldwell JS et al. *JAMA Network Open*.

eTable 2. Parameter Inputs (continued)

	50% Expansion + 100% DDTx			50% Expansion + 25% LDTx			50% Expansion + 50% LDTx			50% Expansion + 100% LDTx			50% Expansion + 200% LDTx		
	Age 18-44	Age 45-64	Age 65-79	Age 18-44	Age 45-64	Age 65-79	Age 18-44	Age 45-64	Age 65-79	Age 18-44	Age 45-64	Age 65-79	Age 18-44	Age 45-64	Age 65-79
CKD, not on waitlist[a]	30.9%	22.5%	28.1%	30.9%	22.5%	28.1%	30.9%	22.5%	28.1%	30.9%	22.5%	28.1%	30.9%	22.5%	28.1%
ESKD, not on waitlist[c]	31.2%	50.0%	61.7%	31.2%	50.0%	61.7%	31.2%	50.0%	61.7%	31.2%	50.0%	61.7%	31.2%	50.0%	61.7%
CKD, on waitlist[c]	8.9%	7.3%	3.1%	8.9%	7.3%	3.1%	8.9%	7.3%	3.1%	8.9%	7.3%	3.1%	8.9%	7.3%	3.1%
ESKD, on waitlist[c]	28.9%	20.2%	7.2%	28.9%	20.2%	7.2%	28.9%	20.2%	7.2%	28.9%	20.2%	7.2%	28.9%	20.2%	7.2%
Progress to ESKD[b]	0.047	0.069	0.072	0.047	0.069	0.072	0.047	0.069	0.072	0.047	0.069	0.072	0.047	0.069	0.072
Add to WL, CKD[c]	0.017	0.021	0.008	0.017	0.021	0.008	0.017	0.021	0.008	0.017	0.021	0.008	0.017	0.021	0.008
Add to WL, ESKD[c]	0.025	0.010	0.003	0.025	0.010	0.003	0.025	0.010	0.003	0.025	0.010	0.003	0.025	0.010	0.003
Remove from WL, CKD[c]	-	-	-	-	-	-	-	-	-	-	-	-	-	-	-
Remove from WL, ESKD[c]	-	-	-	-	-	-	-	-	-	-	-	-	-	-	-
DDTx, CKD[c]	0.004	0.005	0.008	0.004	0.005	0.008	0.004	0.005	0.008	0.004	0.005	0.008	0.004	0.005	0.008
DDTx, ESKD[c]	0.027	0.024	0.031	0.012	0.011	0.015	0.012	0.011	0.014	0.012	0.011	0.014	0.012	0.011	0.014
LDTx, CKD[c]	0.063	0.044	0.045	0.063	0.044	0.045	0.063	0.044	0.045	0.063	0.044	0.045	0.063	0.044	0.045
LDTx, ESKD[c]	0.155	0.122	0.136	0.155	0.121	0.137	0.155	0.121	0.137	0.155	0.121	0.137	0.155	0.121	0.137
New LDTx, CKD	n/a	n/a	n/a	0.001	0.0007	0.0008	0.002	0.001	0.002	0.004	0.003	0.003	0.008	0.005	0.006
New LDTx, ESKD	n/a	n/a	n/a	0.001	0.0007	0.0008	0.002	0.001	0.002	0.004	0.003	0.003	0.008	0.005	0.006
Periop. death[d]	-	-	-	-	-	-	-	-	-	-	-	-	-	-	-
Death, CKD[b]	-	-	-	-	-	-	-	-	-	-	-	-	-	-	-
Waitlist death, ESKD[c]	0.003	0.005	0.004	0.003	0.005	0.004	0.003	0.005	0.004	0.003	0.005	0.004	0.003	0.005	0.004
Death, ESKD, not on waitlist[b]	0.008	0.012	0.018	0.008	0.012	0.018	0.008	0.012	0.018	0.008	0.012	0.018	0.008	0.012	0.018
Death, post-transplant[b]	-	-	-	-	-	-	-	-	-	-	-	-	-	-	-

[a]NHANES, [b]USRDS ADR, [c]SRTR ADR, [d]Ying et al[16]

eTable 2. Parameter inputs. Model inputs are expressed as per-month (per-cycle) probabilities, except for prevalence at model start which is expressed as a percentage. A dash (-) indicates inputs that were unchanged from the status quo under expansion strategies. CKD, chronic kidney disease; ESKD, end-stage kidney

© 2025 Caldwell JS et al. *JAMA Network Open*.

disease; WL, waitlist; NHANES, National Health and Nutrition Examination Survey; USRDS, United States Renal Data System; ADR, Annual Data Report; SRTR, Scientific Registry for Transplant Recipients.

© 2025 Caldwell JS et al. *JAMA Network Open*.

eTable 3. Model Face Validity

A	SRTR Cohort (2016-2022)	Model Output
MWT (months) [75th/25th percentile]	48.2 [21.9–77.2]	48.6 [19.0–101.2]

B	SRTR (2016)	Model Output (Matched to 2016)
DDTx	13,501	13,507
LDTx	5,335	5,346
Waitlist Additions	30,869	30,863
Waitlist Removals	4,411	4,424
Deaths Occurring on the Waitlist	4,830	4,795
Deaths among Dialysis Patients	69,926	69,943

eTable 3. Panel A – Median Wait Time. Panel B – Counts. Model results after modifying parameter inputs to match 2016 rates compared with SRTR data (except deaths among dialysis patients, derived from the USRDS ADR). SRTR, Scientific Registry of Transplant Recipients; MWT, median wait time; DDTx, deceased donor transplant; LDTx, living donor transplant; USRDS, United States Renal Data System.

© 2025 Caldwell JS et al. *JAMA Network Open*.

eFigure. Model Face Validity—Survival Probabilities

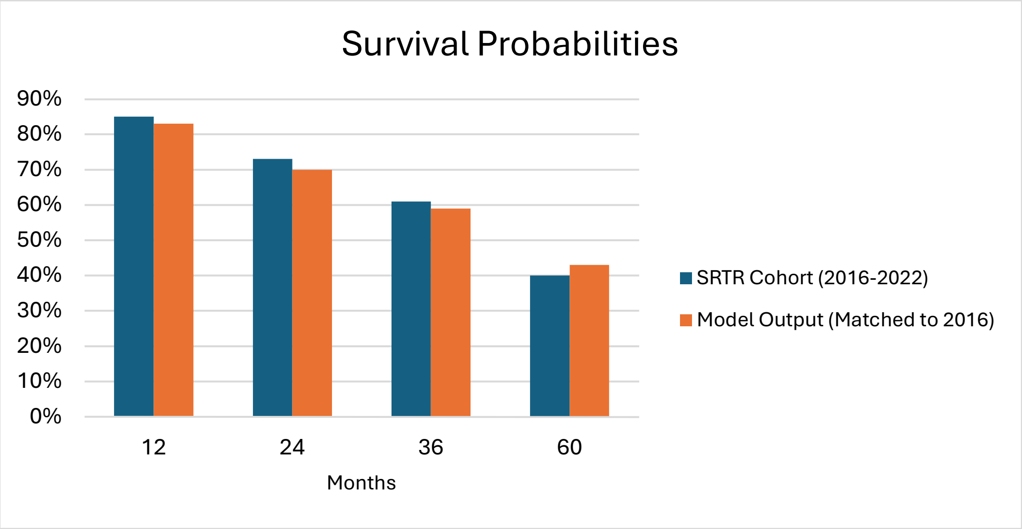

eFigure 1. Survival Probabilities. To compare survival probabilities and wait times between our model and published data, we assembled a cohort listed for kidney-only transplant at any time between January 1, 2016, and August 31, 2022. We counted wait time beginning on the later of two dates: January 1, 2016 or the date of waitlisting. Candidates were censored at waitlist removal, death, or end of follow-up (August 31, 2022). Kaplan-Meier survival analysis was used to compute survival probabilities. We compared these survival probabilities to those produced by our 2016-era model.

© 2025 Caldwell JS et al. *JAMA Network Open*.

eReferences.

1. Centers for Disease Control and Prevention (CDC), National Center for Health Statistics (NCHS), U.S. Department of Health and Human Services, Centers for Disease Control and Prevention. National Health and Nutrition Examination Survey Data (NHANES). Accessed May 12, 2024. https://wwwn.cdc.gov/nchs/nhanes/continuousnhanes/default.aspx?Cycle=2017-2020

2. Leppke S, Leighton T, Zaun D, et al. Scientific Registry of Transplant Recipients: Collecting, analyzing, and reporting data on transplantation in the United States. *Transplant Rev*. 2013;27(2):50-56. doi:10.1016/j.trre.2013.01.002

3. U.S. Department of Health and Human Services, Centers for Disease Control and Prevention. United States Renal Data System (USRDS). Healthy People 2030. Accessed May 28, 2024. https://health.gov/healthypeople/objectives-and-data/data-sources-and-methods/data-sources/united-states-renal-data-system-usrds#:~:text=The%20United%20States%20Renal%20Data,ESRD)%20in%20the%20United%20States

4. Miller WG, Kaufman HW, Levey AS, et al. National Kidney Foundation Laboratory Engagement Working Group Recommendations for Implementing the CKD-EPI 2021 Race-Free Equations for Estimated Glomerular Filtration Rate: Practical Guidance for Clinical Laboratories. *Clin Chem*. 2022;68(4):511-520. doi:10.1093/clinchem/hvab278

5. Ying T, Shi B, Kelly PJ, Pilmore H, Clayton PA, Chadban SJ. Death after Kidney Transplantation: An Analysis by Era and Time Post-Transplant. *J Am Soc Nephrol*. 2020;31(12):2887-2899. doi:10.1681/ASN.2020050566

6. Husain SA, Yu ME, King KL, Adler JT, Schold JD, Mohan S. Disparities in Kidney Transplant Waitlisting Among Young Patients Without Medical Comorbidities. *JAMA Intern Med*. 2023;183(11):1238. doi:10.1001/jamainternmed.2023.5013

7. Schold JD, Srinivas TR, Kayler LK, Meier-Kriesche HU. The Overlapping Risk Profile Between Dialysis Patients Listed and Not Listed for Renal Transplantation. *Am J Transplant*. 2008;8(1):58-68. doi:10.1111/j.1600-6143.2007.02020.x

8. Kochanek KD. *Mortality in the United States, 2019*. U.S. Department of Health and Human Services; Centers for Disease Control and Prevention; National Center for Health Statistics; 2020.

9. Klassen DK, Edwards LB, Stewart DE, Glazier AK, Orlowski JP, Berg CL. The OPTN Deceased Donor Potential Study: Implications for Policy and Practice. *Am J Transplant*. 2016;16(6):1707-1714. doi:10.1111/ajt.13731

10. Israni AK, Zaun DA, Gauntt K, et al. OPTN/SRTR 2022 Annual Data Report: Deceased Organ Donation. *Am J Transplant*. 2024;24(2):S457-S488. doi:10.1016/j.ajt.2024.01.018

11. Mone T, Rosenthal T, Seto T. Direct Measurement of DCD Donor Potential. *Transplantation*. Published online September 5, 2024. doi:10.1097/TP.0000000000005188

12. Marsolais P, Larouche G, Lagacé AM, et al. The Suitability of Potential Organ Donors Using Real Case-Scenarios; Do we Need to Create a “Donor Board” Process for Donors Perceived as Unlikely Suitable? *Transpl Int*. 2022;35:10107. doi:10.3389/ti.2022.10107

13. Goldberg DS, Halpern SD, Reese PP. Deceased Organ Donation Consent Rates Among Racial and Ethnic Minorities and Older Potential Donors*: *Crit Care Med*. 2013;41(2):496-505. doi:10.1097/CCM.0b013e318271198c

© 2025 Caldwell JS et al. *JAMA Network Open*.
