# Supplementary material for: Kidney Transplant Wait Times Under Waiting List Expansion Scenarios
Source: JAMA Netw Open. 2025 Mar 24;8(3):e251665. doi: 10.1001/jamanetworkopen.2025.1665 (PMC11933994; doi:10.1001/jamanetworkopen.2025.1665)
Supplement: Supplement 2. — Data Sharing Statement [file jamanetwopen-e251665-s002.pdf]

Data Sharing Statement

Caldwell. Kidney Transplant Wait Times Under Waiting List Expansion Scenarios. *JAMA Netw Open*. Published March 24, 2025. doi:10.1001/jamanetworkopen.2025.1665

Data
Data available: No

Additional Information
Explanation for why data not available: Patient data requires data use agreement in order to be made available.
